# Research progress in the application of *in situ* hydrogel system in tumor treatment

**DOI:** 10.1080/10717544.2020.1739171

**Published:** 2020-03-13

**Authors:** Weipeng Wei, Hongfang Li, Chengchen Yin, Fushan Tang

**Affiliations:** a Department of Clinical Pharmacy, School of Pharmacy, Zunyi Medical University, Zunyi, China;; b The Key Laboratory of Clinical Pharmacy in Zunyi City, Zunyi, China;; c Key Laboratory of Basic Pharmacology of Ministry of Education and Joint International Research Laboratory of Ethnomedicine of Ministry of Education, Zunyi Medical University, Zunyi, China

**Keywords:** *In situ* hydrogel, tumor treatment, drug delivery system, progress

## Abstract

The *in situ* hydrogel drug delivery system is a hot research topic in recent years. Combining both properties of hydrogel and solution, *in situ* hydrogels can provide many advantages for drug delivery application, including easy application, high local drug concentration, prolonged drug retention time, reduced drug dose *in vivo*, good biocompatibility and improved patient compliance, thus has potential in tumor treatment. In this paper, the related literature reports in recent years were reviewed to summarize and discuss the research progress and development prospects in the application of *in situ* hydrogels in tumor treatment.

## 1. Introduction

Tumors are one type of the malignant diseases that threaten human health most. With the aging of the world’s population and changing in living environment and daily habits, the number of people died from tumors has been increasing dramatically every year, and the world has fallen into conflict with the terrible war of tumors (Shaker et al., 2016; Siegel et al., 2019; Feng et al., 2019). The treatment of tumors has become an important task for medical researchers. At present, the main treatments for tumors include surgical resection, chemotherapy, and radiotherapy (Mao et al., 2016; Watanabe et al., 2017). Among them, chemotherapy is a conventional method and plays an important irreplaceable role in tumor treatment. However, many candidate compounds or drugs currently developed or used clinically in tumor treatment have many problems, including strong hydrophobicity, low bioavailability, instability, greater toxicity and side effects, and lack of targeting, etc., thus fails to fully meet the clinical needs of tumor treatment (Kakinoki et al., 2007; Almeida et al., 2014; Fu & Wu, 2014; Lu et al., 2015; Khaliq et al., 2017; Cullen et al., 2019). Therefore, it is an urgent task to design and construct new delivery system of antitumor drugs for tumor treatment with high efficiency and low toxicity. Sustained and/or targeted release formulations can make the tumor tissues exposed to high-concentration antitumor drugs for a long time, thus avoiding the inconvenience caused by continuous administration, and minimizing the toxic and side effects of antitumor drugs because of relatively low drug uptake by systemic normal cells thanks to tumor local administration (Jung et al., 2018; Chen et al., 2019; Fan et al., 2019; Gibbens-Bandala et al., 2019).

Based on the problems mentioned above, the *in situ* hydrogel drug delivery system has attracted attention from more and more researchers in recent years. Being made of polymer materials with solution, suspension or semi-solid state, the *in-situ* hydrogel system can undergo phase change at the site of administration immediately after administration, making the solution or suspension transformed into a semi-solid or solid state (Chu et al., 2013; Morsi et al., 2016). Due to its liquid nature, it is easy to apply to drug absorption sites and convenient to deliver drugs to wanted sites of patients after the formulation is formed (Paulsamy et al., 2018). Hydrogel formation depends on several factors including pH changes, temperature adjustment and the presence of ions or light. Because of the advantages of this system including local and specific site effects, prolonged drug delivery, reduced drug dosage, increased bioavailability, reduced side effects, and improved patient comfort and compliance (Shen et al., 2012; Lu et al., 2015; Ellah et al., 2018), *in-situ* hydrogels have become a research hotspot for scientific researchers in the treatment of tumors. The researchers in the related fields have tried to develop *in situ* hydrogel system achieving two main goals of drug administration: the first one is to specifically and safely deliver and target the drug to its site of action through local drug delivery (intratumor/paratumor) to maximize its therapeutic efficacy while minimizing its toxicity, based on enhanced antitumor activity with increasing concentration of anti-tumor drugs at the target site (tumor); the second one is to extend the duration of the tumor’s exposure to the drug and maintain its release from the formulated drug carrier to help maintain its therapeutic effect after administration. The *in situ* hydrogel forms a drug depot at the injection site, which may reduce the total number of injections throughout the drug treatment process (Bendas et al., 2013; Moghassemi & Hadjizadeh, 2014; Shaker et al., 2015; Xing et al., 2015, 2019; Omidi et al., 2020). In order to summarize and improve study the application of *in situ* hydrogel system in tumor treatment, this paper focuses on the recent research progress in related areas.

## 2. Types and characteristics of *in situ* hydrogels

**Figure 1.**
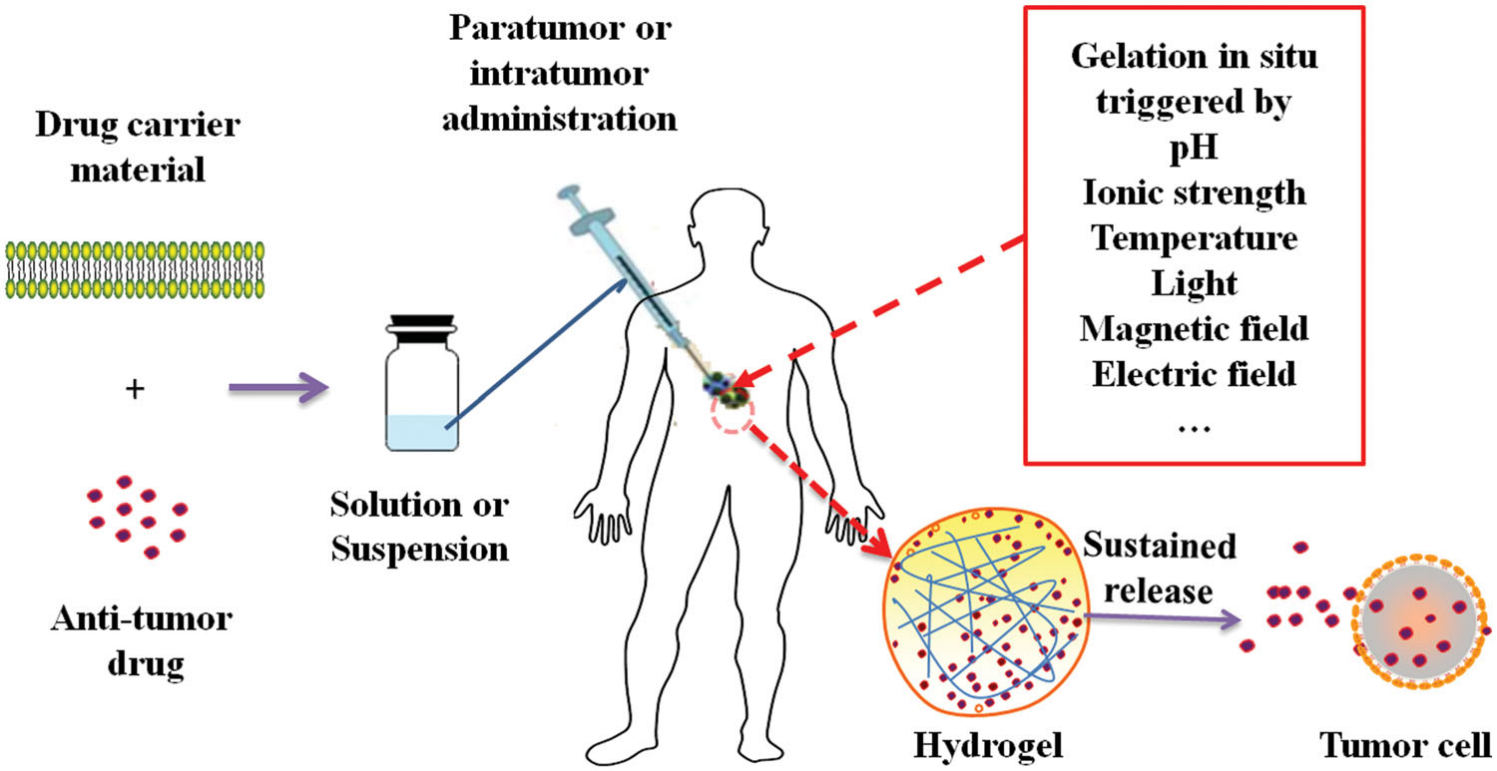
Schematic process of formation of *in situ* hydrogel and sustained release of drugs from the hydrogel into tumor cells.

Because of the benefits from both advantages of solutions and hydrogels, *in situ* hydrogel system has the characteristics of local administration at the disease site, prolonged release cycle, reduced administration dose, and avoiding surgical implantation pain as a novel drug delivery system (Chen et al., 2019; Wei et al., 2019). The *in situ* hydrogel delivery system for anti-tumor drugs before administration is usually in the form of solution or suspension, which was formulated by dissolving or suspending anti-tumor drugs in water solution of drug carrier materials. When the solution or suspension was intratumorally or paratumorly administered, phase transition triggered by external conditions such as pH, temperature, ionic strength, light, magnetic field, and electric field can occur immediately and the hydrogel can form *in situ*. The three-dimensional network structure of the hydrogel can make the anti-tumor drugs incorporated in the *in situ* hydrogel constrained in or near the tumor tissue and sustained released from the hydrogels into tumor cells, thus may improve the anti-tumor effects and limit side-effects of the drugs. The process of formation of *in situ* hydrogel and sustained release of drugs from the *in situ* hydrogel into tumor cells was schematically presented in Figure 1. The advantages of an ideal *in situ* hydrogel as a pharmaceutical preparation can include but not limit to (Cho et al., 2015; Ding et al., 2016; Meng et al., 2019; Zhang et al., 2019): (1) easy to functionalize and clear functional units; (2) biodegradable; (3) good biocompatibility; (4) suitable gelling speed and gel strength; (5) low immunogenicity and toxicity; (6) responsiveness to rapid stimuli from the external environment. With the deepening of researches in related areas, current *in situ* hydrogels can be divided into pH-, temperature-, ionic strength-, temperature-pH-, and light-sensitive and other types. We have consulted the literature on *in situ* hydrogels and their application in anti-tumor drugs for *in vitro* and *in vivo* tumor treatment studies, and summarized their gelling mechanism, drug carrier materials, therapeutic agents, *in vitro* and *in vivo* model into Table 1.

**Table 1.** The *in situ* hydrogel systems in tumor treatment studied in recent years.

Gelling mechanism	Materials	Therapeutic agents	Cancer cell (*in vitro*)	Tumor model (*in vivo*)	References
pH	Hyaluronic acid and fluorescein isothiocyanate conjugated mesoporous silica nanocomposites	Doxorubicin	SKBR3	–	Chen and Liu (2016)
pH	N-carboxyethyl chitosan and dibenzaldehyde-terminated polyethylene glycol	Doxorubicin	HepG2	–	Qu et al. (2017)
pH	Chitosan-grafted dihydrocaffeic acid and oxidized amylopectin	Doxorubicin	HCT116	–	Liang et al. (2019)
pH	Dextran phosphate	Prospidine	HeLa and HepG2	Liver cancer	Solomevich et al. (2019)
Temperature	Polyethylene glycol and monomethoxy poly(ethylene glycol)-poly(caprolactone)	Cisplatin and Paclitaxel	A549	Lung cancer	Wu et al. (2014)
Temperature	Tamoxifen gallate liposomes	Tamoxifen	–	Breast cancer	Shaker et al. (2016)
Temperature	Graphene oxide, folic acid and hyaluronic acid-chitosan-g-poly (N-isopropylacrylamide)	Doxorubicin	MCF-7	Breast cancer	Fong et al. (2017)
Temperature	Negatively charged survivin antisense oligonucleotide and positively charged	Survivin antisense oligonucleotide	MCF-7	Breast cancer	Zhao et al. (2019)
Temperature	Chitosan, β-glyceryl phosphate and polyethylene imine modified superparamagnetic graphene oxide	Doxorubicin	MCF-7	Breast cancer	Zhu et al. (2015)
Temperature	Oxaliplatin and tannic acid polymer nanoparticles	Oxaliplatin and Tannic Acid	HCT26	Colorectal cancer	Ren et al. (2019)
Magnetic field	PEGylated iron oxide nanoparticles	Paclitaxel	–	Breast cancer	Wu et al. (2018)
Magnetic field	Alginate and xanthan gum	Levodopa	SH-SY5Y	–	Kondaveeti et al. (2018)
Magnetic field	α-amino acid residues, CoFe_2_O_4_ and Fe_3_O_4_	Doxorubicin	HeLa	–	Casolaro et al. (2014)
Light	Hemoglobin and polyethylene glycol	Near infrared	A549	Lung cancer	Lee et al. (2018)
Light	Hyaluronic acid, catechol compounds, gallic acid and iron ions	Near infrared	KB, 4T1 and A375	breast cancer, and melanoma	Ko et al. (2019)
Light	Azobenzene and DNA	Doxorubicin	CEM	–	Kang et al. (2011)
Temperature-pH	Poly-N-isopropylacrylamide polymer	Anastrozole	MCF-7	–	Singh et al. (2019)
Temperature-pH	PEG monomethacrylate, and acrylic acid	5-fluorouracil	HepG2	–	Yue et al. (2019)
Temperature-pH	Plunik and polyacrylic acid	Epirubicin	–	Colon adenocarcinoma	Lo et al. (2012)
Temperature-magnetic field	Magnetic iron oxide nanoparticles	Magnetic heat	U87-MG	Glioblastoma	Zhang and Song (2016)
Ion strength/pH	Self-assembling peptide RADA16-I	Emodin	A549 and HepG2	–	Wei et al. (2019)
Ion strength/pH	Self-assembling peptide RADA16-I	Mangiferin	DLD-1 and KYSE 30	–	Meng et al. (2019)
pH-light	Black phosphorus nanosheets, dibenzaldehyde functionalized polymers and polyaspartic hydrazide polymers	Doxorubicin	MDA-MB-231	Breast cancer	Wu et al. (2019)

### 2.1. pH-sensitive *in situ* hydrogel

The molecular skeleton of the materials made of pH-sensitive hydrogel contains a large number of dissociable groups. With the change of the environmental pH and the repulsion between the charges, the molecular chains are stretched, expanded and tangled with each other to form a hydrogel (Yan & Jin, 2012; Sahoo et al., 2013; Shi et al., 2016; Demirdirek & Uhrich, 2017; Rizwan et al., 2017). Such hydrogels usually contain –COOH or –NH_3_, which can form ions with different surrounding pH levels and promote effective shrinkage or expansion of the hydrogel. In the acidic environment, new hydrogen bonds may be formed between the –COOH groups, and the composite hydrogen bonds promote the contraction of the hydrogel and prevent more water from being absorbed, making it difficult to release the loaded drug; while –COOH groups are ionized into carboxyl ions (–COO^−^) under alkaline conditions, and the electrostatic repulsion between the –COO^−^ groups is strengthened, which causes the polymer chain to stretch and then the hydrogel expands and thus water can be absorbed in their 3 D grids, allowing drugs incorporated in the hydrogel to be released quickly. In contrast, the –NH_3_ group shrinks in an alkaline environment, but expands under acidic conditions.

### 2.2. Temperature-sensitive* in situ* hydrogel

Temperature-sensitive *in situ* hydrogels are liquid or semi-solid at room temperature. After administration, as the temperature rises from room temperature to body temperature, a phase transition occurs immediately at the site of application and the liquid or semi-solid form solidifies into hydrogel, resulting in good adhesion and slow release effects (Chu et al., 2013; Lin et al., 2014; Rarokar et al., 2016; Cao et al., 2019).

### 2.3. Ionic strength sensitive* in situ* hydrogel

Ionic strength-sensitive *in situ* hydrogel refers to a kind of hydrogel that can be formed through conformational changes in response to cations such as K^+^, Na^+^, and Ca^2+^ at the administration site. Typical materials used in this kind of hydrogel include alginate, deacetylated gellan gum, etc. When a dilute solution of alginate is introduced to solutions of monovalent or divalent metal ions (K^+^, Na^+^, Ca^2+^), a translucent hydrogel can immediately form *in situ*. Deacetylated gellan gum can immediately form a three-dimensional gel network structure when it encounters monovalent or divalent cations (K^+^, Na^+^, Ca^2+^), thus can also be used *in situ* hydrogel anti-tumor drug delivery system (Rupenthal et al., 2011; Janga et al., 2018; Yang et al., 2018). Due to the good biocompatibility, hydrophilicity and low toxicity related to commonly biogenic nature of drug carrier materials, this type of hydrogel deserves indepth studies and exploitment (Pareek et al., 2017; Shang et al., 2018).

### 2.4. Light-sensitive* in situ* hydrogel

Light-sensitive *in situ* hydrogels are prepared by incorporating photosensitive functional groups into a hydrogel network. In response to light signals, this type of hydrogel can change physical or chemical properties, including viscosity, conductivity, pH, solubility, wettability, mechanical properties, polymer morphology, etc. Under the light stimulus (ultraviolet, infrared, etc.) in the environment, the chromophore undergoes isomerization, cracking or dimerization through the induction of the chromophore in the photoreceptor. Then, the light signals can be transformed into a chemical signal, which in turn affects or changes the structure and performance of this type of hydrogel (Rastogi et al., 2018).

### 2.5. Other types of* in situ* hydrogels

Electric field sensitive *in situ* hydrogel means that the hydrogel can respond and deform quickly when the hydrogel is in an electric field, and the electric field responsive *in situ* hydrogels usually have good water-swelling performance (Kushwaha et al., 2012; Jiang et al., 2019); Magnetic field sensitive *in situ* hydrogel usually consists of a three-dimensional polymeric network and magnetic nanoparticles, and its swelling and shrinking can change with the change of the magnetic field (Liu et al., 2017; Liu et al., 2019).

### 2.6. Dual sensitive* in situ* hydrogel

The double-sensitive *in situ* hydrogel is formed by grafting polymerization of the two sensitive polymers or connecting them with an interpenetrating network, where each polymer chain also independently has different stimulus response (Norouzi et al., 2016; Wang et al., 2017; Fathi et al., 2019). The dual sensitive *in-situ* hydrogels, such as temperature-pH, temperature-ion or pH-magnetic field sensitive *in-situ* hydrogel, not only retain the responsiveness of single sensitive hydrogel, but also have complementary effects on different combinations of them, thus have great potential for increasing applications in various areas.

## 3. Application of *in situ* hydrogel delivery system in tumor treatment

### 3.1. pH-sensitive* in situ* hydrogel

Solid tumors usually show a slightly acidic environment with pH between 6.5 and 7.2. pH-sensitive nano-drug carriers are thus designed to release the drug under the conditions of the tumor’s slightly acidic environment, achieving passively targeting to the tumors (Bai et al., 2018; Wang et al., 2018; Ata et al., 2019; Xie et al., 2018). Injectable hydrogels with tissue adhesion and pH sensitivity are also highly needed for local drug delivery (Wu et al., 2018; Rakhshaei et al., 2019; Solomevich et al., 2019). Chen and Liu (2016) synthesized a mesoporous silica nanocomposite (MSN) of hyaluronic acid (HA) and fluorescein isothiocyanate (FITC), which can self-assemble into hydrogels *in situ* around tumor tissue by pH-responsive interactions (hydrogen bonds) between HA. The hydrogel can indicate the location of the entire tumor and stay in the microenvironment for a long time, providing a wealth of anti-cancer drugs in and around tumor tissue to avoid recurrence. Cytotoxicity experiments showed that FITC-HA-MSN nanohydrogels loaded with doxorubicin (Dox) had increased cytotoxicity to tumor cells with less toxicity to normal cells. Qu et al. (2017) prepared a series of pH-sensitive self-healing (means materials can restore their structures and functionalities after damage) injectable hydrogel by N-carboxyethyl chitosan (CEC) and dibenzaldehyde-terminated polyethylene glycol (PEGDA) synthesized by Michael reaction in aqueous solution. The dynamic covalent Schiff base bond between the amine group of CEC and the benzaldehyde group of PEGDA makes the hydrogel exhibit rapid self-repairing performance. The results of the cytotoxicity test showed that Dox released from the hydrogel matrix significantly inhibited the proliferation of HepG2 cells: at concentrations below 0.1 μg/mL, the hydrogel groups showed a stronger inhibitory effect than the free Dox group; at relatively high levels (0.1 μg/mL and 0.25 μg/mL), the inhibitory effect in the hydrogel group was the same as that in the free Dox group. This indicated that pH-responsive self-healing injectable hydrogels can be excellent candidates for carriers for liver cancer drugs. Liang et al. (2019) developed a series of multifunctional injectable pH-responsive hydrogel based on chitosan-grafted dihydrocaffeic acid (CS-DA) and oxidized amylopectin (OP) via Schiff base reaction. These hydrogels exhibited good injectability, suitable gelation time, *in vitro* pH-dependent equilibrated swelling ratios, morphologies, rheological characteristics, and desirable *in vitro* pH-sensitive drug release behavior. Cell studies showed that the DOX-loaded hydrogel groups had significant inhibitory effect on HCT116 cells better than that in the free DOX groups at same concentration of DOX. This shows that CS-DA/OP hydrogel has great potential as an anti-cancer drug delivery system for colon cancer treatment.

### 3.2. Temperature-sensitive* in situ* hydrogel

Wu et al. (2014) prepared a cisplatin (DDP)- containing thermosensitive poly(ethylene glycol)-poly(ε-caprolactone)-poly(ethylene glycol) (PEG-PCL-PEG/DDP, PECE/DDP) hydrogel and a paclitaxel (PTX)-loaded monomethoxy poly(ethylene glycol)-poly(caprolactone) (MPEG-PCL) micelle to establish *in-situ* hydrogel dual drug delivery system (PEG-PCL-PEG/DDP + MPEG-PCL/PTX, PDMP). PDMP shows a solution state at room temperature and forms a stationary hydrogel at body temperature, making it a promising drug reservoir for orthotopic treatment of lung cancer. *In vivo* lung cancer xenograft model tests showed that PDMP could inhibit tumor growth and prolong survival in tumor-bearing BALB/c nude mice with longer survival time (53 days versus 40 days in the free DDP + PTX group, 26 days in the blank control group and 25 days in the normal saline group). Shaker et al. (2016) prepared tamoxifen citrate liposomes by membrane hydration technology, and incorporated them into Planck’s thermosensitive hydrogel by cold method. Planck’s thermosensitive hydrogel was composed mainly of poloxamer, a kind of polymer nonionic surfactant, polyoxyethylene ether block copolymer, and the gelation temperature of the hydrogel system can be modified to optimal gelation at physiological temperature (34–37 °C) by changing the type and composition of the poloxamer (such as poloxamer 188 or 407). The higher viscosity and elasticity of the hydrogel at physiological temperature than that at room temperature or lower are the keys to controlling the release of the incorporated drug. *In vitro* release data showed the controlled release of tamoxifen (TMC) by the hydrogel for several consecutive days. The final tumor inhibition rates of free tamoxifen, tamoxifen liposomes, and tamoxifen thermosensitive gels were 68.54%, 84.04%, and 97.4%, respectively. The concentrations of tamoxifen in the tumors of mice in the liposome and hydrogel groups were 6 times and 14 times of that in the free drug group, respectively, and the drug concentrations in blood and liver of mice in the liposome and hydrogel groups were both significantly lower than those in the free drug group. The thermosensitive hydrogel immediately formed at body temperature can make the drug concentrated within the tumor site and less transferred to other organs and tissues, thus lessening damage to normal organs and tissues. Fong et al. (2017) prepared the folic acid (FA) conjugated graphene oxide (GO) (GOFA) for targeted delivery of the chemotherapy drug DOX by using the pH-sensitive drug release characteristics of GO after intracellular uptake. GOFA-DOX is further encapsulated in injectable *in situ* formed thermosensitive hyaluronic acid-chitosan-g-poly (N-isopropylacrylamide) (HACPN) hydrogel. Temperature-sensitive HACPN can provide rapid sol-gel phase transition kinetics near the normal temperature of human body to form the hydrogel *in situ*. The gelled HACPN can release drugs continuously to deliver DOX within the tumor. MTT experiments showed that the cytotoxicity of GOFA-DOX/HACPN on MCF-7 cells was dose and time dependents. *In vivo* anti-tumor experiments showed that the intratumor delivery of GOFA DOX/HACPN can be used as a safe and effective drug delivery system for breast cancer chemotherapy.

### 3.3. Magnetic field sensitive* in situ* hydrogel

The local recurrence of tumors after surgical resection is still a major challenge for medical field. Wu et al. (2018) prepared a shear-thinning injectable magnetic hydrogel using an inclusion complex between PEGylated iron oxide (Fe_3_O_4_) nanoparticles and α-cyclodextrin (α-CD). Self-assembling magnetic supramolecular hydrogel (MSH) was designed by partially inclusion complexes with α-CD penetrating the copolymer part on the surface of PEGylated Fe_3_O_4_ nanoparticles, so as to achieve shear thinning injection and controlled thermoreversible gel-sol transition. Experiment results showed that when MSH was evenly injected into the postoperative wound, MSH could flow freely and match the irregular cavity after tumor resection due to the magnetothermal gel-sol transition when exposed to an alternating current magnetic field (ACMF). In addition, biocompatible supramolecular hydrogels could co-load hydrophobic molecules such as PTX into the lipid layer of magnetic nanoparticles (MNPs). The dual structure of MSH can obtain different release processes of multiple drug molecules from a single sample, providing certain concentration of anticancer drugs for a long time. During the gel-sol transition, MNPs-mediated induction heat can exert heat-induced cell damage to tumor cells and trigger the release of anti-tumor drugs. *In vivo* experiments on tumor-bearing animal models showed that MSH has the trustful potential to synergistically eliminate tumors and completely prevent local recurrence of breast cancer after surgical resection. Kondaveeti et al. (2018) prepared a magnetically responsive hydrogel consisting of alginate (Alg) and xanthan gum (XG), which was cross-linked with Ca^2+^ ions and modified by the formation of *in-situ* magnetic nanoparticles (MNP). Compared with magnetic Alg hydrogel, due to the high charge density and molecular weight of XG, magnetic Alg-XG hydrogel had excellent mechanical and swelling properties, which made it significantly more efficient to load levodopa (LD). Under the static electromotive force (EMF) stimulation of 0.4 T, the magnetic stimulus release time of LD from Alg-XG/MNP hydrogel exceeds 30 h, which indicated the potential of sustained release.

### 3.4. Temperature-pH dual-sensitive* in situ* hydrogel

Being sensitive to temperature and pH, poly-N-isopropylacrylamide (PNIPAM) polymer can undergo phase change process at body temperature (37 °C) and/or in slightly acidic environment. Singh et al. (2019) prepared temperature-/pH-triggered PNIPAM smart nanogel systems (NPs) loaded with anastrozole (ANST) by solvent evaporation method for pH and thermally responsive drug delivery. The releases of ANST from this formulation at pH 5.0 were much faster than at pH 7.4 which may indicate ideal release in tumor microenvironment. *In vitro* cytotoxicity showed that the NPs have higher cell uptake, better anti-cancer effects on MCF-7 cells than that of free ANST. Yue et al. (2019) prepared a heat and pH sensitive hydrogel (PAA) through a free-radical copolymerization reaction in water by using PEG monomethacrylate (PEGMA) and acrylic acid (AAc) as monomers, potassium persulfate (K_2_S_2_O_8_), and sodium thiosulfate (Na_2_S_2_O_3_) as initiators. The ideal intestinal adhesion and amphiphilic properties of PEGMA thanks to its microstructure of a hydrophobic polymethacrylate backbone with a hydrophilic PEG side chain can make the hydrogels sensitive to the temperature of the gastrointestinal tract; while the combination of AAc functional groups can make the hydrogels producing stronger physical cross-links through intra-chain and inter-chain associations with non-covalent forces, thus improving the pH sensitivity and mechanical properties of the hydrogels. The dual sensitivity of the PAA hydrogels was confirmed by swelling properties of the hydrogel in different temperatures and buffer solutions being measured by gravimetric analysis. The release profiles of hydrogel-encapsulated 5-fluorouracil (5-Fu) at different pH (stomach pH 1.2 and intestinal pH 7.4) and temperature (25 °C and 37 °C) showed that the release of 5-Fu from the hydrogels was faster at pH 7.4/25 °C than at pH 1.2/37 °C. Cytotoxicity experiments on normal cell lines LO2 and HepG2 cancer cell lines showed that the hydrogel had good cell compatibility and the 5-Fu-loaded hydrogels had a slightly weaker but sustained cell-inhibiting activity than free 5- Fu, which might be due to the slow and long-term release of 5-Fu from drug-loaded hydrogels. Lo et al. developed an oral *in situ* temperature and pH sensitive hydrogel formulation consisting of polyacrylic acid, for the delivery of the anticancer drug epirubicin (Epi). *In vivo* anti-CT-26 mouse colon adenocarcinoma experiments showed better effectiveness of oral administration of Epi *in situ* temperature and pH-sensitive hydrogel preparations in inhibiting tumor growth than that of intravenous or other oral preparations. Generally, *in situ* hydrogels require injection or topical application. This oral *in situ* temperature and pH sensitive hydrogel formulation may provide a promising basis for future use of oral *in situ* hydrogels in cancer treatment.

### 3.5. Light-sensitive* in situ* hydrogel

Lee et al. (2019) prepared a hemoglobin (Hb) hydrogel that exhibited excellent photothermal therapy (PTT) effects *in vitro* and *in vivo* without obvious toxicity observed in major organs. It showed that the therapeutic effect of near infrared (NIR) irradiation on Hb-80 hydrogel was obviously stronger than that without NIR irradiation. Hb hydrogel combined with NIR irradiation is expected to be used in PTT system to improve the anticancer activity of Hb hydrogel loaded drugs. According to relevant reports (Raymond et al., 2015), microorganisms such as bacteria, yeast and fungi can produce iron carriers, which are used as iron chelators for intracellular transport, while carboxyl groups and catechol iron carriers have been proven to have natural iron chelating ability. This indicates that hyaluronic acid (HA) and catechol compound gallic acid (GA) may have iron coordination activity. Therefore, Ko et al. (2019) developed an injectable and photosensitive hydrogel based on the ability of HA and GA to form coordination bonds with iron ions (Fe^3+^). The conjugate of HA and GA (HA-GA) immediately formed a hydrogel in the presence of iron ions, and showed photothermal characteristics in the NIR response. Studies showed that after HA-GA was injected subcutaneously into mice, HA-GA and Fe^3+^ form a hydrogel and stay at the injection site for at least 8 days; HA-GA/Fe hydrogel was injected intratumorally into the mouse, and radiation with NIR showed that NIR irradiation caused complete ablation of tumors in KB cancer cell xenograft mouse and inhibited lung metastasis of 4T1-Luc orthotopic breast tumors. The hydrogel was applied to the skin of an A375 melanoma transplanted tumor, and then subjected to NIR irradiation, which also completely ablated the tumor. The results showed that HA-GA/Fe hydrogel has photothermal anticancer effects on solid tumors and skin cancer. Kang et al. (2011) developed a photoresponsive DNA-cross-linked hydrogel by incorporating photosensitive azobenzene moieties into DNA strands as crosslinkers to make their hybridization to complementary DNAs (cDNAs) responding differently to different wavelengths of light. The hydrogel was utilized for controllable encapsulation and release of fluorescein, horseradish peroxidase, gold nanoparticles and chemotherapy drug doxorubicin by taking advantage of the photoinduced reversible sol-gel conversion. Doxorubicin was encapsulated inside the hydrogel at 450 nm and then released by photons at 350 nm. The *in vitro* release results showed a net drug release rate of 65% within 10 min of doxorubicin with maintained therapeutic effect. This indicated the potential of the hydrogel system to be a promising platform for anti-tumor drug delivery in targeted therapy.

### 3.6. Other types of* in situ* hydrogels

At present, magnetothermal therapy (MHT) has been studied as an effective and noninvasive treatment for cancer. However, the short retention time of magnetic nanoparticles in tumor targets hinders their potential for repeatable treatment. Zhang and Song (2016) developed a biodegradable, heat-sensitive and superparamagnetic iron oxide nanoparticle-supported nanocapsule hydrogel (SPION-NHs) system. SPION-loaded nanocapsule solutions could be converted into hydrogel at body temperature through hydrophobic interactions. MTT results showed that compared to normal MHT, SPION-NHs made U87-MG cells nearly half died after heating at 50 °C for 25 min; confocal microscopy images further verified that cells underwent significant cell death induced by MHT and magnetic thermal ablation (MTA) under an alternating magnetic field (AMF). *In vivo* results from xenograft model showed that after a single injection of SPION-NHs, SPIONs remain in the tumor for more than three weeks, so MHT can be repeated multiple times. The magnatic resonance imaging (MRI) results showed that SPION-NHs was injected into the tumor once and the anti-tumor effect was particularly obvious after four MHT cycles without significant influence on the surrounding normal tissues. Studies showed that classic ion-complementary self-assembling peptide RADA16-I can encapsulate hydrophobic antitumor drugs such as PTX (Liu et al., 2011) and 5-Fu (Ashwanikumar et al., 2016). Recent studies further demonstrated that the peptide RADA16-I and RVDV16-I can stabilize hydrophobic anti-tumor drugs mangiferin and emodin and maintain or enhance their antitumor effects with the suspension-*in situ* hydrogel drug delivery system (Meng et al., 2019; Wei et al., 2019).

### 3.7. FDA approved* in situ* hydrogels

Compared with conventional parenteral and intravenous route of drug administration, the *in situ* hydrogel can be locally injected or targeted to the lesion site, allowing the diseased tissue to be in contact with the drug at a high concentration for a long time, avoiding the inconvenience caused by continuous administration. The local application of *in situ* hydrogel can also limit the toxicity and side effects of the drug (Huang et al., 2016; Demirdirek & Uhrich, 2017; Ailincai et al., 2018). Polyethylene glycol (PEG) (Zhang et al., 2015; Chin et al., 2018), hydroxyethyl methacrylate (HEMA) (Bonifacio et al., 2017), gellan gum (GG) (Boazak et al., 2019), poly-(DL-lactic-co-glycolic acid) (PLGA) (Kempe & Mäder, 2012; Park et al., 2019), lactide/glycolide copolymers and lactide/caprolactone copolymers (Singh et al., 2012) and block copolymers of polyethylene oxide (PEO) and polypropylene oxide (PPO) (Laddha & Mahajan, 2017) have been approved by the US Food and Drug Administration (FDA) as drug carrier materials for *in situ* hydrogel drug delivery system because of their biodegradable properties and nontoxic degradation products. Table 2 contains some of the FDA approved commercially available *in situ* hydrogels. Though only two of them are indicated for cancer, the desirable formulation characteristics and drug delivery performance of these *in situ* hydrogels also indicate that *in-situ* hydrogel can be widely applied to clinical treatment of tumor and other diseases.

**Table 2.** List of some FDA approved *in situ* hydrogels.

Product name	Gelling mechanism	Materials	Active ingredient	Indication	Approved
Atridox	Temperature	PLGA	Doxycycline hyclate	Adult parodontitis	1998
Sandostatin	Temperature	PLGA	Octreotide acetate	Acromegaly	1988
Atrisorb D	Temperature	PLGA	Doxycycline hyclate	Periodontal tissue regeneration	2000
Sublocad	Temperature	PLGA	Buprenorphine	Analgesic	2017
Perseris	Temperature	PLGA	Risperidone	Acute and chronic schizophrenia	2018
Azasite	Temperature	Poloxamer 407	Lidocaine hydrochloric acid	Bacterial conjunctivitis	2007
Lupron depot	pH	PLGA	Leuprolide acetate	Advanced prostate cancer	1995
Eligard	pH	PLGA	Leuprolide acetate	Advanced prostate cancer	2002
Pilopine HS	pH	Carbopol 940	Pilocarpine hydrochloric acid	Glaucoma	1984
Timoptic XE	Ion strength	Gellan gum	Timolol Maleate	Glaucoma	1993

## 4. Conclusions

*In-situ* hydrogels are made of polymer materials in a solution, suspension or semi-solid state, and drugs involving anti-tumor drugs with various properties can be freely loaded into them. After being administered in solution or suspension form, they can immediately undergo phase transformation through different gelling mechanisms at the site of application to form semi-solid or solid hydrogels, which can prolong drug delivery, reduce drug dose, improve bioavailability, and limit side effects of anti-tumor drugs. *In-situ* hydrogel drug delivery systems have been continuously affirmed in tumor treatment research areas and can be successfully applied to various anti-tumor drugs, but most of them are still in the preliminary research stage as a new type of controlled drug delivery system, and the existing problems still need to be further intensively addressed. For example, clinically applied materials for drug carriers require good biocompatibility, biodegradability and relatively cheap material costs. Therefore, research and development on relatively cheap materials with good biocompatibility, biodegradability and appropriate controlled-release performance may be a major task for future research on *in situ* hydrogels, making it a new clinically applicable drug delivery system to contribute to improved clinical treatment of malignant tumors and serve tumor patients better.
